# The association of handgrip strength with all-cause and cardiovascular mortality: results from the National Health and Nutrition Examination Survey database prospective cohort study with propensity score matching

**DOI:** 10.3389/fnut.2023.1183973

**Published:** 2023-09-15

**Authors:** Lijiao Xiong, Zhaohao Zeng, Shuojia Wang, Tingfeng Liao, Xiaohao Wang, Xinyu Wang, Guangyan Yang, Yanchun Li, Lixing Li, Jing Zhu, Pengfei Zhao, Shu Yang, Lin Kang, Zhen Liang

**Affiliations:** ^1^Department of Geriatrics, The Second Clinical Medical College, Jinan University (Shenzhen People’s Hospital), Shenzhen, China; ^2^Guangdong Provincial Clinical Research Center for Geriatrics, Shenzhen Clinical Research Center for Geriatrics, Shenzhen People’s Hospital (The Second Clinical Medical College, Jinan University, The First Affiliated Hospital, Southern University of Science and Technology), Shenzhen, China; ^3^Department of Neurology, Shenzhen People’s Hospital (The Second Clinical Medical College, Jinan University, The First Affiliated Hospital, Southern University of Science and Technology), Shenzhen, China; ^4^Post-Doctoral Scientific Research Station of Basic Medicine, Jinan University, Guangzhou, China

**Keywords:** handgrip strength, all-cause mortality, CVD mortality, NHANES, propensity score-matched analysis

## Abstract

**Objective:**

To investigate the association between handgrip strength (HGS) with all-cause and cardiovascular disease (CVD) mortality in US adults.

**Method:**

We analyzed data from the National Health and Nutrition Examination Survey (NHANES) prospective cohort study (2011–2014) with 10,470 participants. The cox regression analysis, Kaplan–Meier survival curves, fitted curves, ROC curves, and propensity score-matched analysis (PSM) with inverse probability of treatment weighting (IPTW), SMRW (PSM with repeated weights), PA (pairwise algorithm), and OW (overlap weighting) regression analysis were performed to assess the relationship between HGS and all-cause and CVD mortality.

**Results:**

The low HGSs (men <37.4 kg, women <24 kg), was found to be associated with higher all-cause and CVD mortality in a reverse J-shaped curve (*p* < 0.05). Adjusting for multiple covariates including age, BMI, race, education level, marriage status, smoking and alcohol use, and various comorbidities, the hazard ratio (HR) for all-cause mortality in the lowest HGS quintile 1 (Q1) was 3.45 (2.14–5.58) for men and 3.3 (1.88–5.79) for women. For CVD mortality, the HR was 2.99 (1.07–8.37) for men and 10.35 (2.29–46.78) for women. The area under the curve (AUC) for HGS alone as a predictor of all-cause mortality was 0.791 (0.768–0.814) for men and 0.780 (0.752–0.807) for women (*p* < 0.05), while the AUC for HGS and age was 0.851 (0.830–0.871) for men and 0.848 (0.826–0.869) for women (*p* < 0.05). For CVD mortality, the AUC for HGS alone was 0.785 (95% CI 0.738–0.833) for men and 0.821 (95% CI 0.777–0.865) for women (*p* < 0.05), while the AUC for HGS and age as predictors of all-cause mortality was 0.853 (0.861–0.891) for men and 0.859 (0.821–0.896) for women (*p* < 0.05). The HGS Q1 (men <37.4 kg and women <24 kg) was matched separately for PSM. After univariate, multivariate Cox regression models, PSM, IPTW, SMRW, PA, and OW analyses, women had 2.37–3.12 and 2.92–5.12 HRs with low HGS for all-cause and CVD mortality, while men had 2.21–2.82 and 2.33–2.85 for all-cause and CVD mortality, respectively (*p* < 0.05).

**Conclusion:**

Adults with low HGS exhibited a significantly increased risk of both all-cause and CVD mortality, regardless of gender. Additionally, low HGS served as an independent risk factor and predictor for both all-cause and CVD mortality.

## Introduction

The decline in skeletal muscle strength is a common phenomenon associated with aging, which significantly affects physical performance and increases the risk of disability and mortality (1, 2). The assessment of handgrip strength (HGS) serves as a facile and impartial metric of muscle strength and a harbinger of adverse health outcomes (3–5). Research in the past has demonstrated a decrement of 1% *per annum* in HGS beyond midlife (6). The maintenance of high HGS during middle age has been postulated to augment the capacity for healthy aging (7).

Many previous studies had indicated that individuals with low levels of HGS are at an increased risk for mortality and cardiovascular disease (CVD) mortality (3, 7). However, the association between HGS and mortality risk remains controversial, particularly when considering gender differences. A prospective cohort study of individuals aged 75 and above in Taiwan showed no association between HGS and overall or CVD mortality (8). HGS generally tends to be higher in men, often exceeding 10 kilograms. Interestingly, stratified analyses based on gender reveal divergent findings. Some studies report significant associations between lower HGS in both men and women who had higher all-cause and CVD mortality (9). In contrast, a Korean study found that lower HGS in men was significantly associated with all-cause mortality, while not in women (10). Conversely, a study in the UK found an association between HGS and all-cause mortality in women, but not in men (11). Similarly, a study conducted on Japanese patients with type 2 diabetes indicated a significant association between HGS and all-cause mortality in men, whereas not in women (12). Another study conducted within the UK Biobank revealed a correlation between HGS in men and CVD disease mortality, while no significant association was found in women (13).

Given the ongoing debate surrounding the relationship between HGS and mortality for different genders, it is necessary to conduct separate analyses for men and women. HGS is influenced by various factors, including age and comorbidities (14), which may confound the relationship between HGS and mortality. A study across 28 countries showed that low HGS in older adults, regardless of gender, was associated with all-cause mortality (5). However, this study only included general demographic data as confounding factors and did not consider the impact of comorbidities. Propensity score matching (PSM) analysis is a valuable approach that can effectively match individuals across different groups, controlling for confounding factors and reducing selection bias, thereby enabling more robust causal inferences (15). Therefore, this study aims to conduct separate analyses for males and females of HGS and its association with all-cause and CVD mortality among the adult population in the United States. By utilizing propensity score matching, we hope to minimize the impact of confounding factors and obtain more reliable results.

## Materials and methods

### Data sources and preparation

The population was from the National Health and Nutrition Examination Survey (NHANES, 2011–2014), which is a comprehensive cross-sectional survey conducted across all 50 states and the District of Columbia in the United States (16). Informed consent was obtained from all participants after approval by the Institutional Review Board of the National Center for Health Statistics (NCHS) (16). Through a multilevel stratified probability design, 5,000 participants were sampled annually, and each performed a standardized questionnaire and physical examination. This representative survey’s data have been published online every 2 years since 1999. Online data sets are accessible for public use at https://www.cdc.gov/nchs/nhanes/index.htm (16). Given that this study utilized publicly available deidentified data and waived informed consent, the institutional review board at the Shenzhen People’s Hospital deemed the study exempt. All research findings were reported in compliance with STROBE guidelines throughout.

**Figure 1:**
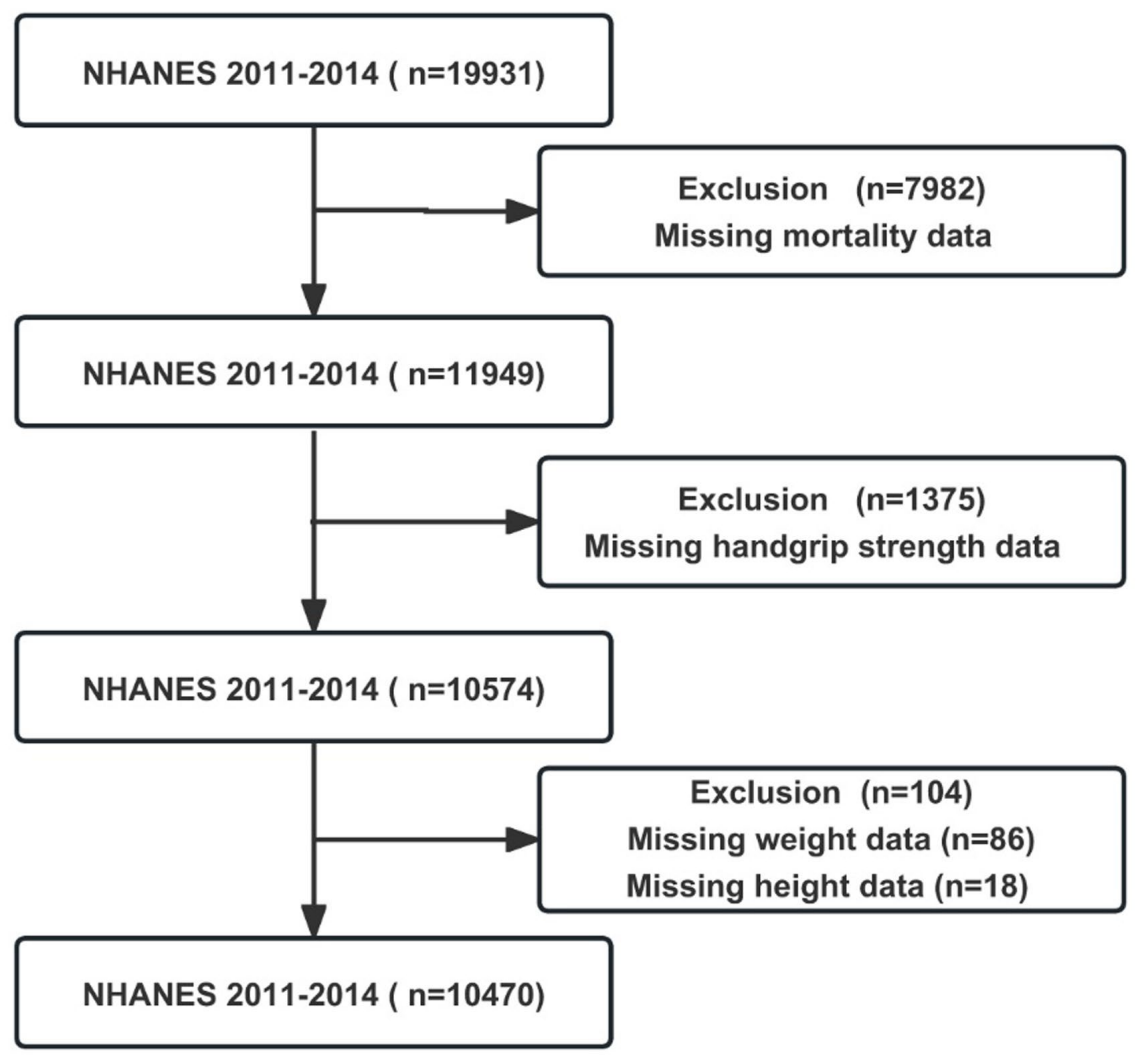
The flowchart of participants in NHANSE 2011–2014.

This research used publicly accessible NHANES data from 2011–2014, including 19,931 individuals. The following exclusion criteria were used to restrict our analysis: missing data for mortality (*n* = 7,982); missing data for HGS (*n* = 1,375); missing data for weight (*n* = 86); missing data for height (*n* = 18). In the final, 10,470 participants were enrolled in this research (Figure 1).

### Handgrip strength

In this study, muscle strength estimation was achieved by utilizing HGS data from both hands, as detailed in the NHANES Muscle Strength—Grip Test protocol (16). Participants were instructed to firmly hold the dynamometer with either their dominant or non-dominant hand while exhaling to avoid raising intrathoracic pressure, unless physically unable to stand. During the measurement process, the procedure was explained and demonstrated to the participant by trained examiners, and the grip size of the dynamometer was adjusted to the participant’s hand size. Testing was conducted three times on the same hand alternately. In this study, the HGS was defined as the highest value obtained using either hand.

### All-cause and CVD mortality

The all-cause mortality rate was the number of participants who died from any cause after the date of the baseline survey but before December 31, 2018 (16). Data on mortality follow-up using the ICD-10 from the NHANES are publicly available in the Public-use Linked Mortality Files.^1^ The CVD death was classified as ICD 054-068.

### Covariates

A variety of clinical and demographic factors were considered as covariates., including age, sex, BMI, race and ethnicity, educational level, marital status, diabetes, asthma, congestive heart failure, coronary heart disease, stroke, cancer, high blood pressure, smoking status, and alcohol drinking status. In NHANES, these informations were collected from survey responses. Participants were classified as Mexican American, other Hispanic, non-Hispanic Asian, White, Black, or Other (including multiracial). The education level of participants was categorized as Less than 9th grade, 9th–11th grade (includes 12th grade with no diploma), High school graduate or equivalent, college graduate or above, and others. The categories for marital status were described as married, widowed, divorced, separated, never married, living with a partner, and other. Asthma, congestive heart failure, coronary heart disease, stroke, cancer, high blood pressure, and diabetes were diagnosed by a physician or other health professional. The smoking and drinking behaviors were categorized as never, past, and current use. The formula for calculating BMI as weight (kg)/[height (m^2^) × height (m^2^)].

### Statistical analysis

For continuous variables, 95% confidence intervals (CIs) were provided, while for categorical variables, percentage frequencies were provided. *T*-tests and χ^2^ tests were used to compare continuous and categorical data. No imputation approach was applied because all variables had low missing data rates. The mortality risk is calculated using Cox proportional hazards regression models. Curve fitting of restricted cubic spline plot and Kaplan–Meier curves are visually illustrated. HGS alone and models that included HGS and age were used as predictors for mortality. An analysis of propensity score matching (PSM) with the following covariates was conducted to reduce potential selection bias. After calculating individual propensity scores using a Cox regression model, the nearest-neighbor matching algorithm with a caliper width of 0.2 standard deviations of the propensity score was utilized to match patients among the lowest HGS and other groups. We then utilized four distinct weighting techniques: the inverse probability of treatment weighting (IPTW) regression analysis (14), the standardized mortality ratio weighting (SMRW) regression analysis (14), the pairwise algorithm (PA) weighted regression analysis (5) and the overlay weight (OW) regression analysis (15). Statistical analyses were carried out using the *R* software package (http://www.R-project.org, The R Foundation) and the Free Statistics software version 1.7. Statistical significance was determined by a two-sided *p*-value <0.05.

## Results

### Demographics

The final sample included 10,470 participants in the study, with 5,175 (49.4%) men and 5,295 (50.6%) women with a mean age of 37.1 ± 11.6 years (Table 1). There were 1,230 patients with diabetes mellitus (11.7%), 1,595 patients with asthma (15.2%), 308 patients with congestive hypertension (2.9%), 366 patients with coronary heart disease (3.5%), 339 patients with stroke (3.2%), 886 patients with cancer (8.5%), and 3,604 patients with high blood pressure (34.4%). The mean follow-up period was 81.2 (range 70–95) months for all causes and cause-specific mortality. The follow-up period ended with 847 (8.1%) deaths, 222 (26.2%) deaths from cardiovascular, 201 (23.7%) deaths from cancer, 45 (5.3%) deaths from chronic respiratory diseases, 35 (4.1%) deaths from accidents, 48 (5.7%) deaths from cerebrovascular disease, 15 (1.8%) deaths from Alzheimer’s disease, 40 (4.7%) deaths from diabetes mellitus, 14 (1.7%) deaths from influenza and pneumonia, 22 (2.6%) deaths from renal disease, and 205 (24.2%) deaths from all other causes.

**Table 1:** Baseline characteristics of participants in NHANES 2011–2014.

Characteristic	Total (*n* = 10,470)	Men (*n* = 5,175)	Women (*n* = 5,295)	*p*-value
**Handgrip strength (kg)**	37.1 ± 11.6	45.4 ± 9.7	29.0 ± 6.3	<0.001
**Age, mean ± SD**	46.9 ± 18.4	46.7 ± 18.5	47.2 ± 18.4	0.144
**Race, n (%)**				0.133
Mexican American	1222 (11.7)	621 (12)	601 (11.4)	
Other Hispanic	978 (9.3)	449 (8.7)	529 (10)	
Non-Hispanic White	4196 (40.1)	2082 (40.2)	2114 (39.9)	
Non-Hispanic Black	2480 (23.7)	1216 (23.5)	1264 (23.9)	
Non-Hispanic Asian	1268 (12.1)	632 (12.2)	636 (12)	
Other race including multiracial	326 (3.1)	175 (3.4)	151 (2.9)	
**Education, n (%)**				<0.001
Less than 9th grade	782 (7.5)	421 (8.1)	361 (6.8)	
9–11th grade	1560 (14.9)	804 (15.5)	756 (14.3)	
High school graduate or equivalent	2355 (22.5)	1228 (23.7)	1127 (21.3)	
College graduate or above	5768 (55.1)	2720 (52.6)	3048 (57.6)	
Other	5 (0.0)	2 (0)	3 (0.1)	
**Marriage, n (%)**				<0.001
Married	4930 (47.1)	2641 (51)	2289 (43.2)	
Widowed	725 (6.9)	181 (3.5)	544 (10.3)	
Divorced	1092 (10.4)	439 (8.5)	653 (12.3)	
Separated	337 (3.2)	139 (2.7)	198 (3.7)	
Never married	2042 (19.5)	1072 (20.7)	970 (18.3)	
Living with partner	758 (7.2)	408 (7.9)	350 (6.6)	
Other	586 (5.6)	295 (5.7)	291 (5.5)	
**Drinking, n (%)**				<0.001
Never drinking	1567 (16.1)	491 (10.1)	1076 (22)	
Current drinking	6992 (71.8)	3996 (82.4)	2996 (61.3)	
Ever drinking	1178 (12.1)	364 (7.5)	814 (16.7)	
**Smoking, n (%)**				<0.001
Never smoking	6010 (57.5)	2519 (48.7)	3491 (66)	
Current smoking	2130 (20.4)	1243 (24)	887 (16.8)	
Ever smoking	2317 (22.2)	1407 (27.2)	910 (17.2)	
**Diabetes, n (%)**				0.853
No	9240 (88.3)	4564 (88.2)	4676 (88.3)	
Yes	1230 (11.7)	611 (11.8)	619 (11.7)	
**Asthma, n (%)**				<0.001
No	8875 (84.8)	4487 (86.7)	4388 (82.9)	
Yes	1595 (15.2)	688 (13.3)	907 (17.1)	
**Congestive heart failure, n (%)**				0.581
No	10162 (97.1)	5018 (97)	5144 (97.1)	
Yes	308 (2.9)	157 (3)	151 (2.9)	
**Coronary heart disease, n (%)**				<0.001
No	10104 (96.5)	4943 (95.5)	5161 (97.5)	
Yes	366 (3.5)	232 (4.5)	134 (2.5)	
**Stroke, n (%)**				0.345
No	10131 (96.8)	5016 (96.9)	5115 (96.6)	
Yes	339 (3.2)	159 (3.1)	180 (3.4)	
**Cancer, n (%)**				0.05
No	9584 (91.5)	4765 (92.1)	4819 (91)	
Yes	886 (8.5)	410 (7.9)	476 (9)	
**High blood pressure, n (%)**				0.062
No	6866 (65.6)	3439 (66.5)	3427 (64.7)	
Yes	3604 (34.4)	1736 (33.5)	1868 (35.3)	
**Weight (kg), mean ± SD**	81.0 ± 21.9	86.2 ± 21.1	75.9 ± 21.5	<0.001
**Height (cm), mean ± SD**	167.5 ± 10.1	174.3 ± 7.7	160.8 ± 7.2	<0.001
**BMI, mean ± SD**	28.8 ± 7.1	28.3 ± 6.2	29.3 ± 7.8	<0.001
**All-cause mortality, n (%)**				<0.001
No	9623 (91.9)	4706 (90.9)	4917 (92.9)	
Yes	847 (8.1)	469 (9.1)	378 (7.1)	
**Follow-up time, mean ± SD**	81.2 ± 17.8	81.1 ± 18.4	81.3 ± 17.3	0.56
**Cause of death, n (%)**				0.098
CVD mortality	222 (26.2)	116 (24.7)	106 (28)	
Cancer mortality	201 (23.7)	104 (22.2)	97 (25.7)	
Chronic respiratory disease mortality	45 (5.3)	20 (4.3)	25 (6.6)	
Accidents mortality	35 (4.1)	25 (5.3)	10 (2.6)	
Cerebrovascular disease mortality	48 (5.7)	31 (6.6)	17 (4.5)	
Alzheimer’s disease mortality	15 (1.8)	6 (1.3)	9 (2.4)	
Diabetes mellitus mortality	40 (4.7)	25 (5.3)	15 (4)	
Influenza and pneumonia mortality	14 (1.7)	6 (1.3)	8 (2.1)	
Kidney disease mortality	22 (2.6)	12 (2.6)	10 (2.6)	
All other causes of mortality	205 (24.2)	124 (26.4)	81 (21.4)	

### The relationship between HGS and all-cause mortality or CVD mortality

**Figure 2:**
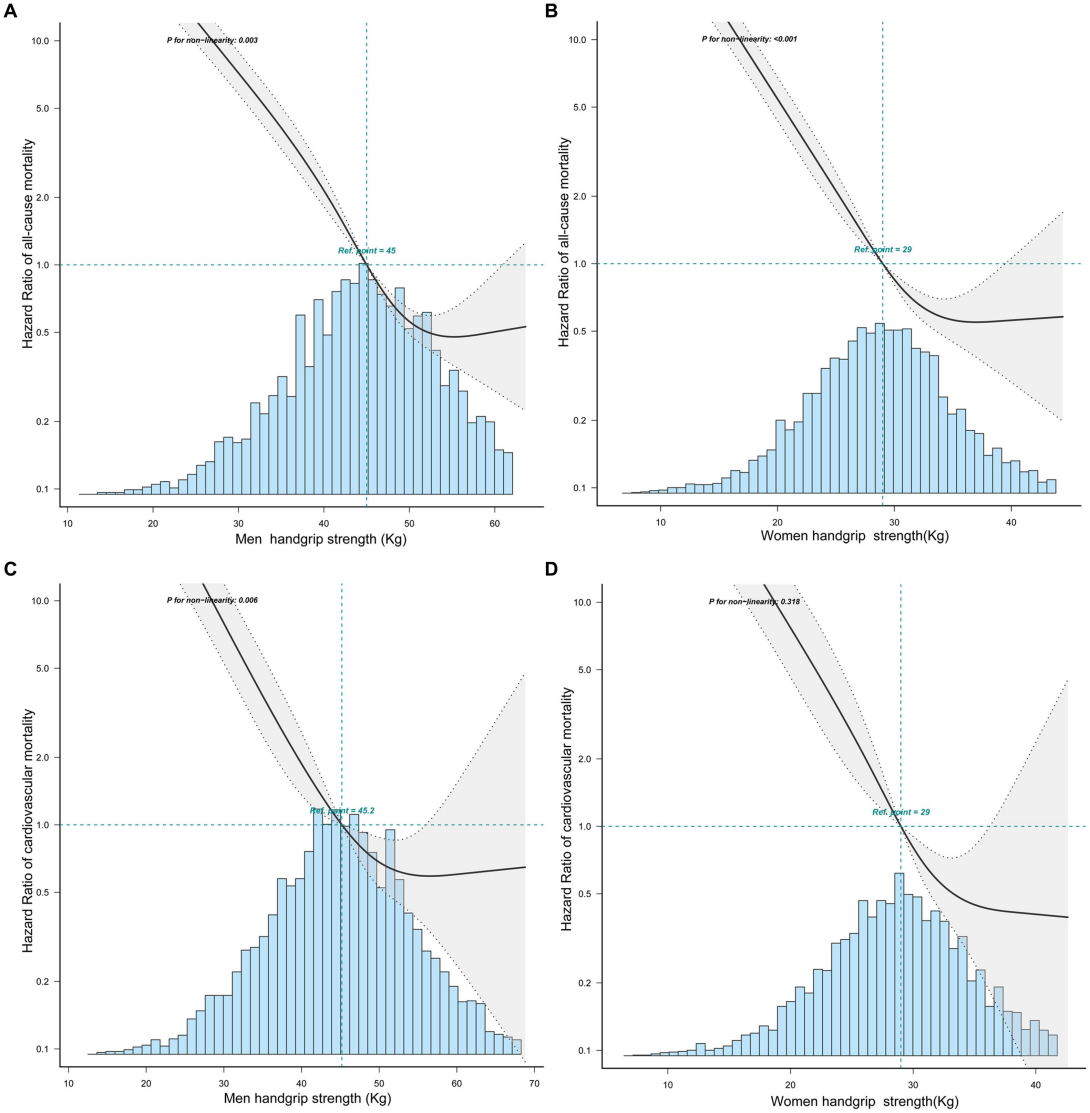
The relationship between handgrip strength with all-cause and CVD mortality in men and women by curve fitting. **(A)** The relationship between HGS and all-cause mortality in men. **(B)** The relationship between HGS and all-cause mortality in women. **(C)** The relationship between HGS and CVD mortality in men. **(D)** The relationship between HGS and CVD mortality in women.

**Figure 3:**
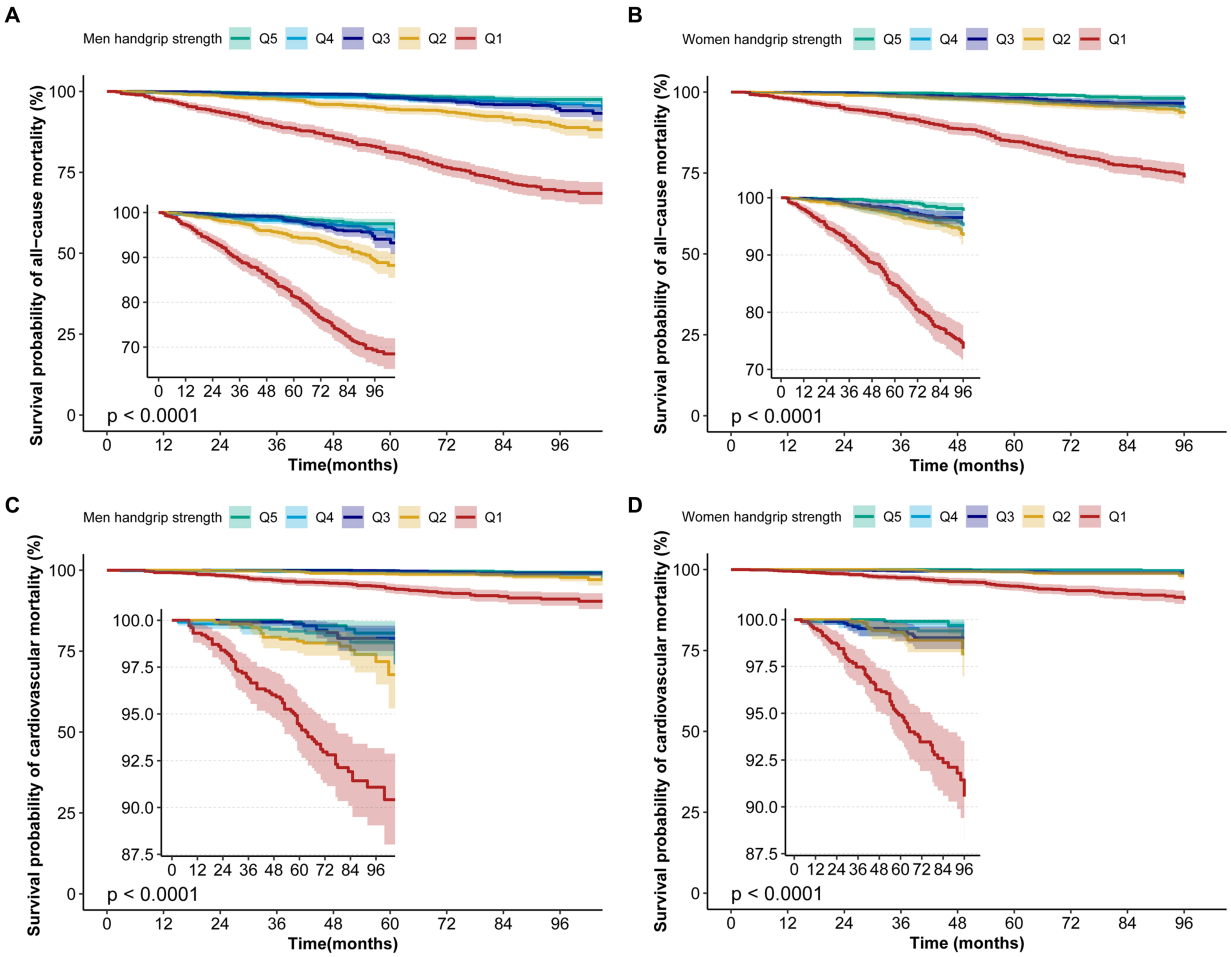
Kaplan–Meier survival curves for HGS associated with all-cause and CVD mortality risk. **(A)** The Kaplan–Meier survival curves for HGS with all-cause mortality in men. **(B)** The Kaplan–Meier survival curves for HGS with all-cause mortality in women. **(C)** The Kaplan–Meier survival curves for HGS with CVD mortality in men. **(D)** The Kaplan–Meier survival curves for HGS with CVD mortality in women.

According to curve fitting, men and women with higher HGS had a lower risk of all-cause mortality, approaching the reverse J shape (*p* < 0.05) (Figure 2). For CVD mortality, similar reverse J-shaped curves were observed between HGS and CVD mortality, with a significant difference for men (*p* < 0.05) but not for women (*p* > 0.05) (Figure 2). Kaplan–Meier survival curves indicated that low HGS (men <37.4 kg, women <24 kg) was associated with an increased all-cause and CVD mortality risk (*p <* 0.05) (Figure 3).

The results from multivariable Cox regression analyses are presented in Table 2. Compared to Q5, the unadjusted hazard ratio (HR) of HGS Q1 for all-cause mortality (model 1) was 14.19 (95% CI 9.28–21.71) for men and 15.88 (95% CI 9.71–25.98) for women. The HR for CVD mortality was 17.44 (95% CI 7.05–43.13) for men and 42.18 (95% CI 10.35–171.82) for women (model 1). After adjusting for age and BMI (model 2), the HR of HGS Q1 for all-cause mortality was 3.39 (95% CI 2.13–5.39) for men and 3.22 (95% CI 1.88–5.53) for women, whereas for CVD mortality it was 3.13 (95% CI 1.16–8.39) for men and 9.59 (95% CI 2.21–41.63) for women. Further adjustment for age, BMI, race, education level, marriage status, drinking, and smoking (model 3), the HR of HGS Q1 for all-cause mortality was 3.85 (95% CI 2.39–6.19) for men and 3.78 (95% CI 2.16–6.6) for women, whereas for CVD mortality it was 3.9 (95% CI 1.42–10.76) for men and 12.36 (95% CI 2.77–55.21) for women. Finally, after adjusting for age, BMI, race, education level, marriage status, drinking, smoking, asthma, congestive heart failure, coronary heart disease, stroke, cancer, high blood pressure, and diabetes (model 4), the HR of HGS Q1 for all-cause mortality was 3.45 (95% CI 2.14–5.58) for men and 3.3 (95% CI 1.88–5.79) for women, whereas for CVD mortality was 2.99 (95% CI 1.07–8.37) for men and 10.35 (95% CI 2.29–46.78) for women. All of the unadjusted and adjusted HR for HGS Q1 were statistically significant (*p < 0.05*), regardless of gender.

**Table 2:** Association of handgrip strength with all-cause and CVD mortality.

HGS	Deaths (%)	Model 1	*p*-value	Model 2	*p*-value	Model 3	*p*-value	Model 4	*p*-value
**Men all-cause mortality**									
Q5	23 (2.2)	1 (Reference)		1 (Reference)		1 (Reference)		1 (Reference)	
Q4	34 (3.3)	1.47 (0.87–2.5)	0.15	1.11 (0.65–1.89)	0.705	1.14 (0.67–1.94)	0.639	1.1 (0.65–1.88)	0.717
Q3	44 (4.2)	1.91 (1.15–3.17)	0.012	0.99 (0.59–1.65)	0.969	1.05 (0.63–1.77)	0.84	1.02 (0.6–1.71)	0.952
Q2	87 (8.5)	3.95 (2.5–6.26)	<0.001	1.49 (0.92–2.41)	0.102	1.68 (1.03–2.73)	0.037	1.56 (0.96–2.54)	0.076
Q1	281 (27.2)	14.19 (9.28–21.71)	<0.001	3.39 (2.13–5.39)	<0.001	3.85 (2.39–6.19)	<0.001	3.45 (2.14–5.58)	<0.001
Trend. test	469 (9.1)	2.21 (2.03–2.42)	<0.001	1.52 (1.38–1.67)	<0.001	1.56 (1.41–1.72)	<0.001	1.51 (1.36–1.67)	<0.001
**Women all-cause mortality**									
Q5	17 (1.6)	1 (Reference)		1 (Reference)		1 (Reference)		1 (Reference)	
Q4	39 (3.8)	2.41 (1.36–4.26)	0.002	1.7 (0.96–3.02)	0.07	1.81 (1.01–3.24)	0.045	1.76 (0.98–3.15)	0.057
Q3	34 (3.2)	1.98 (1.11–3.55)	0.021	1.11 (0.61–2)	0.733	1.31 (0.72–2.39)	0.371	1.28 (0.7–2.33)	0.418
Q2	51 (4.9)	3.15 (1.82–5.45)	<0.001	1.2 (0.68–2.13)	0.526	1.48 (0.83–2.65)	0.187	1.38 (0.77–2.47)	0.284
Q1	237 (22.4)	15.88 (9.71–25.98)	<0.001	3.22 (1.88–5.53)	<0.001	3.78 (2.16–6.6)	<0.001	3.3 (1.88–5.79)	<0.001
Trend. test	378 (7.1)	2.15 (1.95–2.37)	<0.001	1.37 (1.23–1.52)	<0.001	1.4 (1.25–1.56)	<0.001	1.34 (1.2–1.5)	<0.001
**Men CVD mortality**									
Q5	5 (0.5)	1 (Ref)		1 (Ref)		1 (Ref)		1 (Ref)	
Q4	11 (1.1)	2.2 (0.76–6.32)	0.145	1.54 (0.53–4.44)	0.429	1.7 (0.59–4.95)	0.328	1.58 (0.54–4.59)	0.403
Q3	8 (0.8)	1.6 (0.52–4.9)	0.408	0.7 (0.23–2.2)	0.544	0.74 (0.23–2.4)	0.615	0.68 (0.21–2.22)	0.524
Q2	17 (1.7)	3.56 (1.31–9.64)	0.013	1.07 (0.38–3.04)	0.895	1.34 (0.46–3.85)	0.591	1.19 (0.41–3.45)	0.745
Q1	75 (7.3)	17.44 (7.05–43.13)	<0.001	3.13 (1.16–8.39)	0.024	3.9 (1.42–10.76)	0.008	2.99 (1.07–8.37)	0.037
Trend. test	116 (2.2)	2.31 (1.93–2.78)	<0.001	1.48 (1.21–1.81)	<0.001	1.54 (1.24–1.9)	<0.001	1.41 (1.14–1.75)	0.002
**Women CVD mortality**									
Q5	2 (0.2)	1 (Ref)		1 (Ref)		1 (Ref)		1 (Ref)	
Q4	7 (0.7)	3.68 (0.76–17.71)	0.104	2.74 (0.57–13.27)	0.21	3.43 (0.71–16.72)	0.127	3.12 (0.64–15.23)	0.16
Q3	10 (0.9)	4.96 (1.09–22.63)	0.039	3.02 (0.65–13.99)	0.158	4.1 (0.87–19.19)	0.074	3.77 (0.8–17.72)	0.093
Q2	13 (1.3)	6.85 (1.55–30.37)	0.011	2.83 (0.62–12.92)	0.179	4.05 (0.87–18.81)	0.074	3.53 (0.76–16.51)	0.109
Q1	74 (7)	42.18 (10.35–171.82)	<0.001	9.59 (2.21–41.63)	0.003	12.36 (2.77–55.21)	0.001	10.35 (2.29–46.78)	0.002
Trend. test	106 (2)	2.72 (2.2–3.38)	<0.001	1.73 (1.38–2.18)	<0.001	1.73 (1.37–2.19)	<0.001	1.66 (1.31–2.11)	<0.001

Model 1: crude model. Model 2: adjusted for age and BMI. Model 3: adjusted for age, BMI, race, education level, marriage status, drinking, and smoking. Model 4: adjusted for age, BMI, race, education level, marriage status, drinking, smoking, asthma, congestive heart failure, coronary heart disease, stroke, cancer, high blood pressure, and diabetes.

### The ROC curves for HGS and age in predicting all-cause and CVD mortality

**Figure 4:**
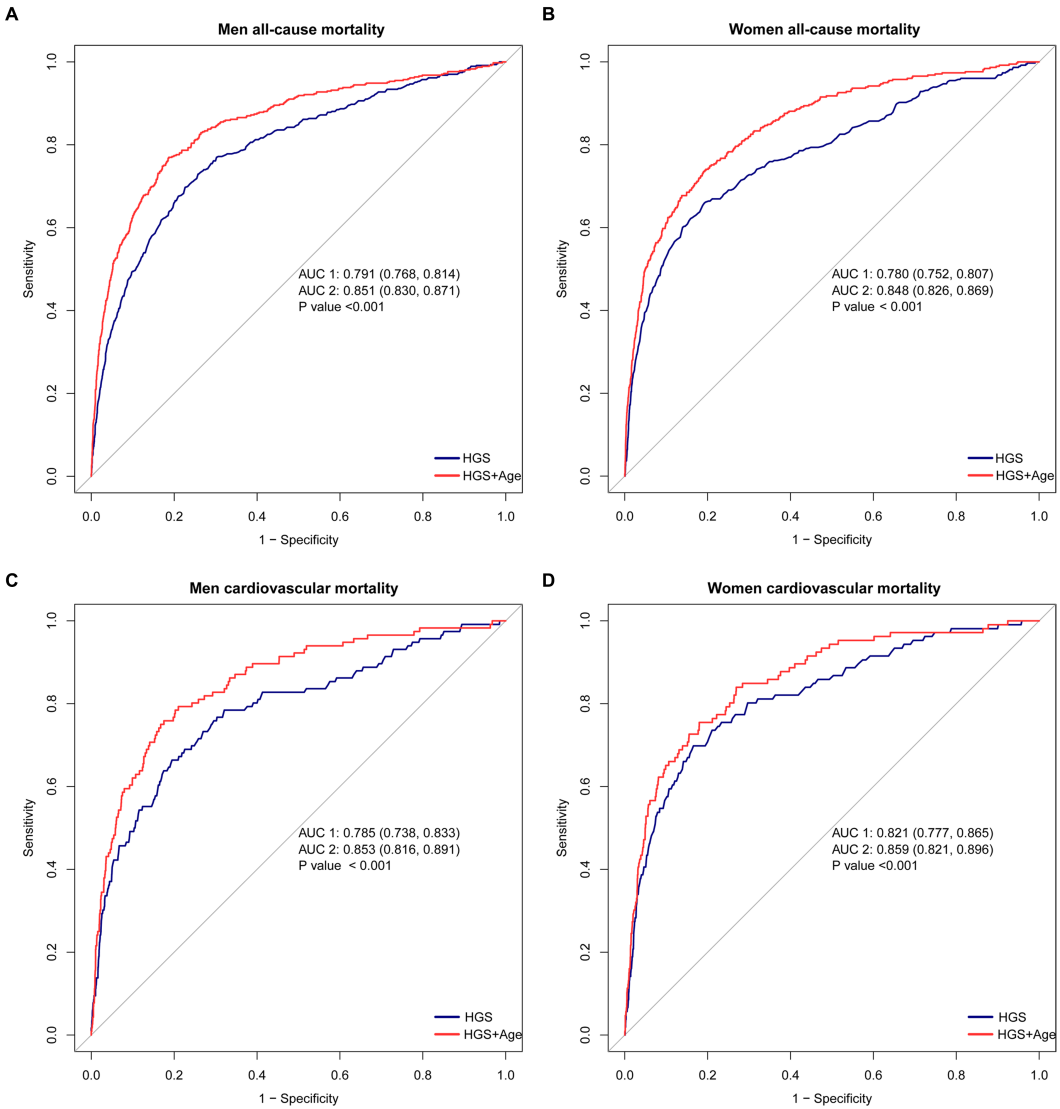
The ROC curves for HGS and age in predicting all-cause and CVD mortality. **(A)** The ROC curves for HGS and age in predicting all-cause mortality in men. **(B)** The ROC curves for HGS and age in predicting all-cause mortality in women. **(C)** The ROC curves for HGS and age in predicting CVD mortality in men. **(D)** The ROC curves for HGS and age in predicting CVD mortality in women. ^##^The blue lines represent the model using HGS as a single predictive factor, while the red lines represent the model using both HGS and Age as predictive factors.

HGS shows significant predictive capacity for both all-cause and CVD mortality. When HGS is considered as a standalone predictor, it exhibits notable predictive power. In men, the area under the curve (AUC) for HGS in predicting all-cause mortality is 0.791 (95% CI 0.768–0.814) (blue line in Figure 4A). A similar trend is observed in women, with an AUC of 0.780 (95% CI 0.752–0.807) (blue line in Figure 4B) (*p* < 0.05).

Furthermore, HGS demonstrates robust predictive capability for CVD mortality. In men, the AUC for HGS as a predictor is 0.785 (95% CI 0.738–0.833) (blue line in Figure 4C). Likewise, women display strong predictive ability, with an AUC of 0.821 (95% CI 0.777–0.865) (blue line in Figure 4D) (*p* < 0.05).

The predictive accuracy of all-cause mortality can be significantly enhanced by combining HGS with age. When HGS and age are jointly considered, the AUC improves substantially. In men, the AUC for this joint prediction increases to 0.851 (95% CI 0.830–0.871) (red line in Figure 4A). Similarly, in women, the AUC rises to 0.848 (95% CI 0.826–0.869) (red line in Figure 4B) (*p* < 0.05).

Moreover, the combination of HGS and age showcases remarkable performance in predicting CVD mortality. In men, the AUC for this combination rises to 0.853 (95% CI 0.861–0.891) (red line in Figure 4C). Similarly, in women, an outstanding AUC of 0.859 (95% CI 0.821–0.896) is displayed (red line in Figure 4D) (*p* < 0.05).

### The HRs with low HGS for all-cause and CVD mortality with PSM, IPTW, SMRW, PA, and OW analysis

**Figure 5:**
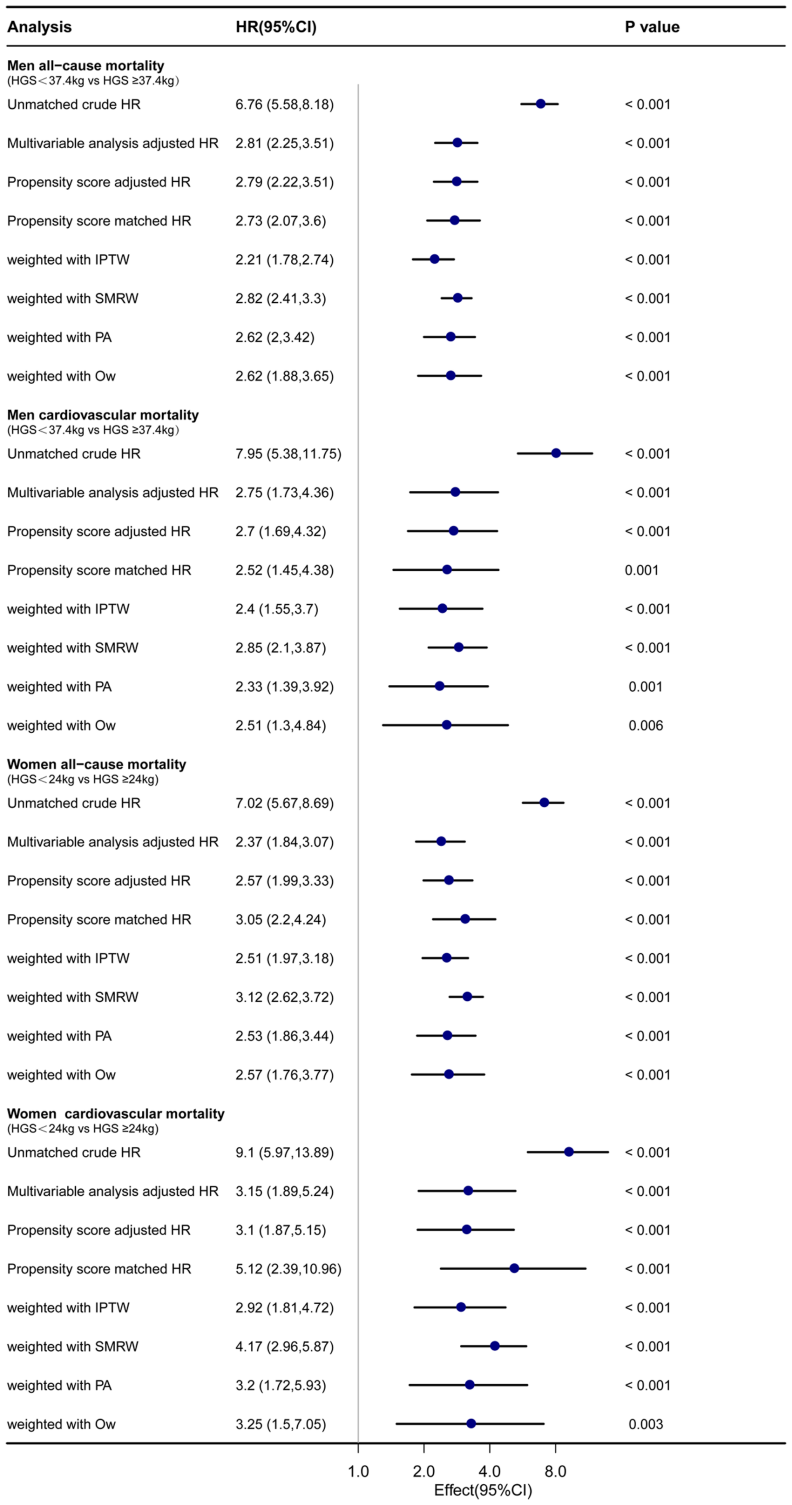
Forest plot shows HRs of all-cause and CVD mortality in men and women with low HGS using a variety of models. IPTW: the inverse probability of treatment weighting regression analysis. SMRW: the standardized mortality ratio weighting regression analysis. PA: pairwise algorithm weighted regression analysis. OW: overlay weight regression analysis.

The lowest quintile of HGS (men <37.4 kg and women <24 kg) was matched as a separate group for PSM. After PSM, the number of male participants decreased from 1,034 to 774, and the number of female participants decreased from 1,059 to 788. There were no statistically significant differences in baseline characteristics after PSM, including age, BMI, race, education, marital status, smoking, alcohol use, and comorbidities, between the low HGS group and the high HGS group among both males and females (Table 1; Supplementary Figure S1). In both men and women with low HGS (men <37.4 kg and women <24 kg), there were significantly higher hazard ratios (HRs) for all-cause and CVD mortality by various sensitivity analyses (*p* < 0.05). For men with low HGS, the HRs for all-cause and CVD mortality ranged from 2.21 to 2.82 and 2.33 to 2.85, respectively (*p* < 0.05). For women with low HGS, the HRs for all-cause and CVD mortality ranged from 2.37 to 3.12 and 2.92 to 5.12, respectively (*p* < 0.05) (Figure 5).

## Discussion

Low HGS has been associated with adverse health outcomes. However, there is still ongoing debate about gender differences in this relationship between HGS and mortality (10–12). Our study revealed a reverse J-shape association between HGS and death from all-causes and CVD disease. The results indicated that men and women with lower HGS faced higher risks of both all-cause and CVD mortality, even after rigorous adjustments for demographic factors and comorbidities. Moreover, the group differences and the impact of confounding factors were minimized by PSM, IPTW, SMRW, PA, and OW weighting. These methods yielded robust results: low HGS significantly predicts all-cause and CVD mortality both in men and women. Our finding further confirms the importance of HGS as an independent risk factor in predicting all-cause and CVD mortality.

The findings of this research have significant implications for public health. Lower HGS is closely linked to increased risks of mortality and CVD, indicating the importance of maintaining optimal muscle strength for overall health (14, 17–20). The underlying mechanisms behind this association are multifaceted. HGS serves as a surrogate marker for early-stage sarcopenia (21), physical fitness, and functional capacity, with lower HGS potentially indicating reduced physical activity levels, elevated inflammation, and compromised cardiovascular and metabolic function (18, 20, 22–25). Additionally, low handgrip strength may reflect an overall decline in muscle mass and strength, which is frequently associated with age-related conditions and an elevated risk of adverse health outcomes (26–28).

Compared to previous studies, this research conducted separate analyses for men and women, recognizing potential gender differences. Inconsistencies in previous research results may stem from several factors. Firstly, variations in the selected study populations, including differences in age, gender, race, and geographical distribution, could contribute to divergent findings (6). Secondly, disparities in study design and methodology, such as sample size, duration of study, statistical approaches, and adjustments for potential confounding factors, may influence the consistency of results (5, 18, 20). Additionally, discrepancies may arise from variances in the instruments and standardized procedures used for grip strength measurements across studies (20). Furthermore, it is important to consider potential gender differences and confounding variables (7). Additionally, the study employed PSM techniques, minimizing the impact of confounding variables and strengthening the validity of the results. By utilizing PSM, this study overcomes some limitations of prior research designs.

The study has several strengths, including large sample size, a long follow-up period, and adjustment for various potential confounders. Limitations inherent in our investigation include the absence of data concerning alterations in HGS over time. Moreover, despite adjusting for a broad range of confounding variables, it remains possible that there exist additional unmeasured factors that influence the results. Additionally, as our study solely comprised American adults, our findings may not be universally applicable to other populations. Consequently, future research should center on longitudinal studies, which can examine the link between HGS and mortality risk across time and in heterogeneous populations. Despite these limitations, the findings of this study have important clinical implications. HGS is a simple and noninvasive measure that can be easily obtained in clinical settings. Identifying individuals with low HGS may allow earlier interventions to prevent or manage chronic diseases and ultimately improve health outcomes.

In conclusion, our study highlights the importance of low HGS as an independent risk factor and a significant predictor for all-cause and CVD mortality. These findings emphasized the importance of maintaining HGS as a potential means of reducing mortality risk and suggested that low HGS may serve as a valuable predictor of mortality risk, with potential implications for clinical practice and public health interventions.

## Data availability statement

The original contributions presented in the study are included in the article/Supplementary material, further inquiries can be directed to the corresponding authors. Publicly available datasets were analyzed in this study. The NHANES data can be found at: https://www.cdc.gov/nchs/nhanes/index.htm.

## Ethics statement

The National Center for Health Statistics (NCHS) Institutional Review Board approved all study procedures, and participants provided written informed consent. This representative survey’s data have been published online every two years since 1999. Online data sets are accessible for public use at https://www.cdc.gov/nchs/nhanes/index.htm. Because the study used publicly available deidentified data and informed consent was waived. Based on a publicly accessible database, this study did not require ethical approval or informed consent. The patients/participants provided their written informed consent to participate in this study.

## Author contributions

LX and ZZ: conceiving the protocol, data analysis and interpretation, acquisition of data, statistical analysis and interpretation of data, and manuscript preparation. SW, TL, XHW, and XYW: study concept and design, statistical analysis, and interpretation of data. GY, YL, LL, JZ, and PZ: data analysis and data acquisition. SY, LK, and ZL: concept and design, final drafting of the manuscript, and study supervision. All authors contributed to the article and approved the submitted version.

## Funding

This research was supported by grants from the Science and Technology Planning Project of Shenzhen City, Guangdong Province, China (Nos. KCXFZ20201221173600001 and JCYJ20220818102605013).

## Conflict of interest

The authors declare that the research was conducted in the absence of any commercial or financial relationships that could be construed as a potential conflict of interest.

## Publisher’s note

All claims expressed in this article are solely those of the authors and do not necessarily represent those of their affiliated organizations, or those of the publisher, the editors and the reviewers. Any product that may be evaluated in this article, or claim that may be made by its manufacturer, is not guaranteed or endorsed by the publisher.

## Footnotes

^1^https://www.cdc.gov/nchs/data-linkage/mortality.htm

## Supplementary material

SUPPLEMENTARY FIGURE 1 “Absolute standardized differences before and after propensity score matching comparing covariates for participants with and without low HGS in men (HGS < 37.4Kg) and women (HGS < 24 kg).”
