# Supplementary figures and images for: The association of handgrip strength with all-cause and cardiovascular mortality: results from the National Health and Nutrition Examination Survey database prospective cohort study with propensity score matching
Source: Front Nutr. 2023 Sep 15;10:1183973. doi: 10.3389/fnut.2023.1183973 (PMC10541216; doi:10.3389/fnut.2023.1183973)

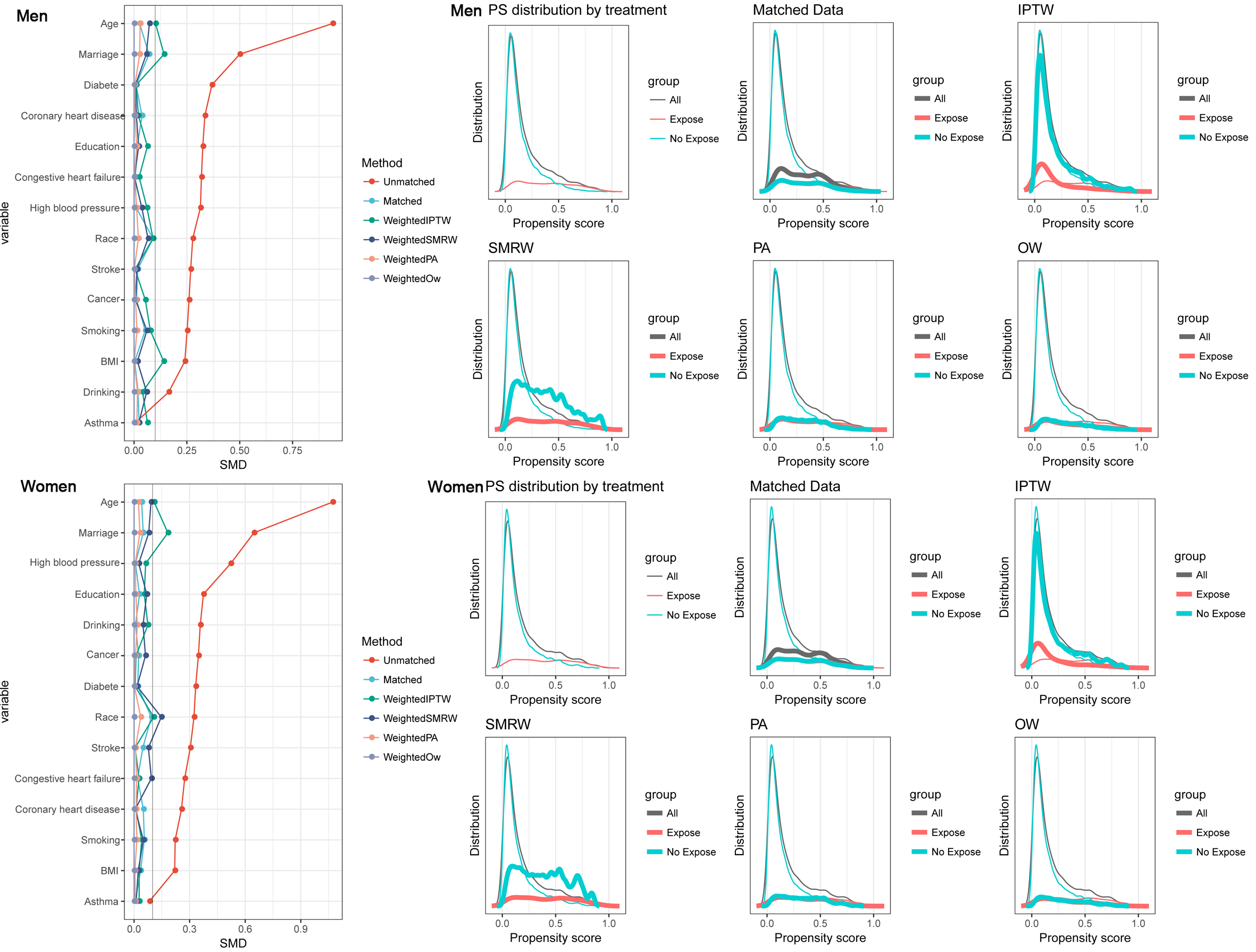

Supplement: SUPPLEMENTARY FIGURE 1 — “Absolute standardized differences before and after propensity score matching comparing covariates for participants with and without low HGS in men (HGS < 37.4Kg) and women (HGS < 24 kg).” [file Image_1.TIF]
