# Supplementary material for: The association of handgrip strength with all-cause and cardiovascular mortality: results from the National Health and Nutrition Examination Survey database prospective cohort study with propensity score matching
Source: Front Nutr. 2023 Sep 15;10:1183973. doi: 10.3389/fnut.2023.1183973 (PMC10541216; doi:10.3389/fnut.2023.1183973)
Supplement: Supplementary file 2 [file Table_1.docx]

**Supplementary Table 1 The characteristics of participants before and after Propensity-Score–Matched in men and women**

| **characteristics** |  | | **Unmatched Men** | |  |  | **Propensity-Score–Matched Men** | | |  | **Unmatched Women** | | |  | **Propensity-Score–Matched Women** | | |
| --- | --- | --- | --- | --- | --- | --- | --- | --- | --- | --- | --- | --- | --- | --- | --- | --- | --- |
|  |  | | **HGS≥37.4Kg**  **(n = 4141)** | **HGS＜37.4Kg**  **(n = 1034)** | ***P value*** |  | **HGS≥37.4Kg**  **(n = 774)** | **HGS＜37.4Kg**  **(n = 774)** | ***P value*** |  | **HGS≥24Kg**  **(n = 4236)** | **HGS＜24Kg**  **(n = 1059)** | ***P value*** |  | **HGS≥24Kg**  **(n = 788)** | **HGS＜24Kg**  **(n = 788)** | ***P value*** |
|  |  |  |  |  |  |  |  |  |  |  |  |  |  |  |  |  |  |
| handgrip, Mean ± SD | | 48.8 ± 7.4 | | 31.7 ± 4.6 | < 0.001 |  | 45.4 ± 6.3 | 32.3 ± 4.4 | < 0.001 |  | 31.2 ± 4.8 | 20.2 ± 3.2 | < 0.001 |  | 29.0 ± 3.9 | 20.7 ± 2.8 | < 0.001 |
| Age, Mean ± SD | | 43.3 ± 16.8 | | 60.3 ± 18.8 | < 0.001 |  | 56.0 ± 17.6 | 56.5 ± 18.9 | 0.606 |  | 43.5 ± 16.5 | 62.0 ± 18.0 | < 0.001 |  | 58.2 ± 16.0 | 58.9 ± 18.2 | 0.415 |
| BMI, Mean ± SD | | 28.6 ± 6.3 | | 27.1 ± 5.8 | < 0.001 |  | 27.4 ± 4.8 | 27.4 ± 6.1 | 0.95 |  | 29.6 ± 8.0 | 28.0 ± 6.9 | < 0.001 |  | 28.7 ± 6.3 | 28.5 ± 7.3 | 0.486 |
| Race, n (%) |  | | |  | < 0.001 |  |  |  | 0.682 |  |  |  | < 0.001 |  |  |  | 0.637 |
| Mexican American |  | | 489 (11.8) | 132 (12.8) |  |  | 111 (14.3) | 104 (13.4) |  |  | 476 (11.2) | 125 (11.8) |  |  | 113 (14.3) | 90 (11.4) |  |
| Other Hispanic |  | | 339 (8.2) | 110 (10.6) |  |  | 68 (8.8) | 82 (10.6) |  |  | 401 (9.5) | 128 (12.1) |  |  | 95 (12.1) | 97 (12.3) |  |
| Non-Hispanic White |  | | 1661 (40.1) | 421 (40.7) |  |  | 321 (41.5) | 303 (39.1) |  |  | 1656 (39.1) | 458 (43.2) |  |  | 300 (38.1) | 321 (40.7) |  |
| Non-Hispanic Black |  | | 1043 (25.2) | 173 (16.7) |  |  | 130 (16.8) | 141 (18.2) |  |  | 1108 (26.2) | 156 (14.7) |  |  | 137 (17.4) | 134 (17) |  |
| Non-Hispanic Asian |  | | 458 (11.1) | 174 (16.8) |  |  | 117 (15.1) | 122 (15.8) |  |  | 469 (11.1) | 167 (15.8) |  |  | 124 (15.7) | 126 (16) |  |
| Other Race including |  | | 151 (3.6) | 24 (2.3) |  |  | 27 (3.5) | 22 (2.8) |  |  | 126 (3) | 25 (2.4) |  |  | 19 (2.4) | 20 (2.5) |  |
| MultiRacial |  |  |  |  |  |  |  |  |  |  |  |  |  |  |  |  |  |
| Education, n (%) |  | | |  | < 0.001 |  |  |  | 0.997 |  |  |  | < 0.001 |  |  |  | 0.952 |
| Less than 9th grade |  | | 253 (6.1) | 168 (16.2) |  |  | 99 (12.8) | 97 (12.5) |  |  | 206 (4.9) | 155 (14.6) |  |  | 89 (11.3) | 86 (10.9) |  |
| 9-11th grade |  | | 644 (15.6) | 160 (15.5) |  |  | 119 (15.4) | 122 (15.8) |  |  | 584 (13.8) | 172 (16.2) |  |  | 118 (15) | 120 (15.2) |  |
| High school graduate or equivalent |  | | 1001 (24.2) | 227 (22) |  |  | 177 (22.9) | 174 (22.5) |  |  | 881 (20.8) | 246 (23.2) |  |  | 191 (24.2) | 183 (23.2) |  |
| College graduate or above |  | | 2242 (54.1) | 478 (46.2) |  |  | 378 (48.8) | 380 (49.1) |  |  | 2564 (60.5) | 484 (45.7) |  |  | 390 (49.5) | 399 (50.6) |  |
| other |  | | 1 (0) | 1 (0.1) |  |  | 1 (0.1) | 1 (0.1) |  |  | 1 (0) | 2 (0.2) |  |  |  |  |  |
| Marriage, n (%) |  | | |  | < 0.001 |  |  |  | 0.925 |  |  |  | < 0.001 |  |  |  | 0.987 |
| Married |  | | 2084 (50.3) | 557 (53.9) |  |  | 419 (54.1) | 428 (55.3) |  |  | 1871 (44.2) | 418 (39.5) |  |  | 339 (43) | 353 (44.8) |  |
| Widowed |  | | 75 (1.8) | 106 (10.3) |  |  | 52 (6.7) | 43 (5.6) |  |  | 258 (6.1) | 286 (27) |  |  | 144 (18.3) | 145 (18.4) |  |
| Divorced |  | | 335 (8.1) | 104 (10.1) |  |  | 82 (10.6) | 83 (10.7) |  |  | 511 (12.1) | 142 (13.4) |  |  | 119 (15.1) | 118 (15) |  |
| Separated |  | | 106 (2.6) | 33 (3.2) |  |  | 27 (3.5) | 29 (3.7) |  |  | 162 (3.8) | 36 (3.4) |  |  | 25 (3.2) | 25 (3.2) |  |
| Never married |  | | 896 (21.6) | 176 (17) |  |  | 137 (17.7) | 140 (18.1) |  |  | 867 (20.5) | 103 (9.7) |  |  | 94 (11.9) | 86 (10.9) |  |
| Living with partner |  | | 377 (9.1) | 31 (3) |  |  | 25 (3.2) | 26 (3.4) |  |  | 310 (7.3) | 40 (3.8) |  |  | 33 (4.2) | 31 (3.9) |  |
| other |  | | 268 (6.5) | 27 (2.6) |  |  | 32 (4.1) | 25 (3.2) |  |  | 257 (6.1) | 34 (3.2) |  |  | 34 (4.3) | 30 (3.8) |  |
| Drinking, n (%) |  | | |  | < 0.001 |  |  |  | 0.932 |  |  |  | < 0.001 |  |  |  | 0.988 |
| Never drinking |  | | 363 (9.3) | 128 (13.4) |  |  | 85 (11) | 85 (11) |  |  | 756 (19.4) | 320 (32.6) |  |  | 224 (28.4) | 222 (28.2) |  |
| Current drinking |  | | 3261 (83.6) | 735 (77.2) |  |  | 629 (81.3) | 625 (80.7) |  |  | 2526 (64.7) | 470 (47.9) |  |  | 411 (52.2) | 414 (52.5) |  |
| Ever drinking |  | | 275 (7.1) | 89 (9.3) |  |  | 60 (7.8) | 64 (8.3) |  |  | 622 (15.9) | 192 (19.6) |  |  | 153 (19.4) | 152 (19.3) |  |
| Smoking, n (%) |  | | |  | < 0.001 |  |  |  | 0.585 |  |  |  | < 0.001 |  |  |  | 0.545 |
| Never smoking |  | | 2052 (49.6) | 467 (45.2) |  |  | 359 (46.4) | 340 (43.9) |  |  | 2769 (65.4) | 722 (68.3) |  |  | 543 (68.9) | 523 (66.4) |  |
| Current smoking |  | | 1053 (25.5) | 190 (18.4) |  |  | 149 (19.3) | 161 (20.8) |  |  | 774 (18.3) | 113 (10.7) |  |  | 94 (11.9) | 99 (12.6) |  |
| Ever smoking |  | | 1031 (24.9) | 376 (36.4) |  |  | 266 (34.4) | 273 (35.3) |  |  | 688 (16.3) | 222 (21) |  |  | 151 (19.2) | 166 (21.1) |  |
| Diabetes, n (%) |  | | |  | < 0.001 |  |  |  | 0.846 |  |  |  | < 0.001 |  |  |  | 0.692 |
| No |  | | 3764 (90.9) | 800 (77.4) |  |  | 629 (81.3) | 626 (80.9) |  |  | 3842 (90.7) | 834 (78.8) |  |  | 646 (82) | 652 (82.7) |  |
| Yes |  | | 377 (9.1) | 234 (22.6) |  |  | 145 (18.7) | 148 (19.1) |  |  | 394 (9.3) | 225 (21.2) |  |  | 142 (18) | 136 (17.3) |  |
| Asthma, n (%) |  | | |  | 0.723 |  |  |  | 0.642 |  |  |  | 0.033 |  |  |  | 0.89 |
| No |  | | 3587 (86.6) | 900 (87) |  |  | 676 (87.3) | 682 (88.1) |  |  | 3487 (82.3) | 901 (85.1) |  |  | 664 (84.3) | 666 (84.5) |  |
| Yes |  | | 554 (13.4) | 134 (13) |  |  | 98 (12.7) | 92 (11.9) |  |  | 749 (17.7) | 158 (14.9) |  |  | 124 (15.7) | 122 (15.5) |  |
| Congestive heart failure, n (%) |  | | | | < 0.001 |  |  |  | 0.91 |  |  |  | < 0.001 |  |  |  | 0.7 |
| No |  | | 4075 (98.4) | 943 (91.2) |  |  | 732 (94.6) | 733 (94.7) |  |  | 4163 (98.3) | 981 (92.6) |  |  | 755 (95.8) | 758 (96.2) |  |
| Yes |  | | 66 (1.6) | 91 (8.8) |  |  | 42 (5.4) | 41 (5.3) |  |  | 73 (1.7) | 78 (7.4) |  |  | 33 (4.2) | 30 (3.8) |  |
| Coronary heart disease, n (%) |  | | |  | < 0.001 |  |  |  | 0.46 |  |  |  | < 0.001 |  |  |  | 0.321 |
| No |  | | 4023 (97.2) | 920 (89) |  |  | 706 (91.2) | 714 (92.2) |  |  | 4171 (98.5) | 990 (93.5) |  |  | 750 (95.2) | 758 (96.2) |  |
| Yes |  | | 118 (2.8) | 114 (11) |  |  | 68 (8.8) | 60 (7.8) |  |  | 65 (1.5) | 69 (6.5) |  |  | 38 (4.8) | 30 (3.8) |  |
| Stroke, n (%) |  | | |  | < 0.001 |  |  |  | 0.735 |  |  |  | < 0.001 |  |  |  | 0.347 |
| No |  | | 4060 (98) | 956 (92.5) |  |  | 731 (94.4) | 734 (94.8) |  |  | 4152 (98) | 963 (90.9) |  |  | 746 (94.7) | 754 (95.7) |  |
| Yes |  | | 81 (2) | 78 (7.5) |  |  | 43 (5.6) | 40 (5.2) |  |  | 84 (2) | 96 (9.1) |  |  | 42 (5.3) | 34 (4.3) |  |
| Cancer, n (%) |  | | |  | < 0.001 |  |  |  | 0.817 |  |  |  | < 0.001 |  |  |  | 0.619 |
| No |  | | 3882 (93.7) | 883 (85.4) |  |  | 676 (87.3) | 679 (87.7) |  |  | 3950 (93.2) | 869 (82.1) |  |  | 668 (84.8) | 675 (85.7) |  |
| Yes |  | | 259 (6.3) | 151 (14.6) |  |  | 98 (12.7) | 95 (12.3) |  |  | 286 (6.8) | 190 (17.9) |  |  | 120 (15.2) | 113 (14.3) |  |
| High blood pressure, n (%) |  | | |  | < 0.001 |  |  |  | 0.837 |  |  |  | < 0.001 |  |  |  | 0.88 |
| No |  | | 2875 (69.4) | 564 (54.5) |  |  | 446 (57.6) | 450 (58.1) |  |  | 2961 (69.9) | 466 (44) |  |  | 390 (49.5) | 393 (49.9) |  |
| Yes |  | | 1266 (30.6) | 470 (45.5) |  |  | 328 (42.4) | 324 (41.9) |  |  | 1275 (30.1) | 593 (56) |  |  | 398 (50.5) | 395 (50.1) |  |
| All-cause mortality, n (%) |  | | |  | < 0.001 |  |  |  | < 0.001 |  |  |  | < 0.001 |  |  |  | < 0.001 |
| No |  | | 3953 (95.5) | 753 (72.8) |  |  | 704 (91) | 597 (77.1) |  |  | 4095 (96.7) | 822 (77.6) |  |  | 740 (93.9) | 648 (82.2) |  |
| Yes |  | | 188 (4.5) | 281 (27.2) |  |  | 70 (9) | 177 (22.9) |  |  | 141 (3.3) | 237 (22.4) |  |  | 48 (6.1) | 140 (17.8) |  |
| Heart mortality, n (%) |  | | |  | < 0.001 |  |  |  | 0.002 |  |  |  | < 0.001 |  |  |  | < 0.001 |
| No |  | | 4100 (99) | 959 (92.7) |  |  | 756 (97.7) | 732 (94.6) |  |  | 4204 (99.2) | 985 (93) |  |  | 780 (99) | 749 (95.1) |  |
| Yes |  | | 41 (1) | 75 (7.3) |  |  | 18 (2.3) | 42 (5.4) |  |  | 32 (0.8) | 74 (7) |  |  | 8 (1) | 39 (4.9) |  |
| Follow-up time, Mean ± SD | | 83.0 ± 16.0 | | 73.8 ± 24.4 | < 0.001 |  | 82.1 ± 17.2 | 77.0 ± 22.9 | < 0.001 |  | 82.8 ± 15.3 | 75.6 ± 22.8 | < 0.001 |  | 81.6 ± 15.7 | 78.7 ± 21.3 | 0.002 |
