# Supplementary material for: The association of handgrip strength with all-cause and cardiovascular mortality: results from the National Health and Nutrition Examination Survey database prospective cohort study with propensity score matching
Source: Front Nutr. 2023 Sep 15;10:1183973. doi: 10.3389/fnut.2023.1183973 (PMC10541216; doi:10.3389/fnut.2023.1183973)
Supplement: Supplementary file 3 [file Table_1.pdf]

Supplementary Table 1 The characteristics of participants before and after Propensity-Score–Matched in men and women

**characteristics**	**Unmatched Men**			**Propensity-Score–Matched Men**			**Unmatched Women**			**Propensity-Score–Matched Women**		
	**HGS≥37.4Kg (n = 4141)**	**HGS＜37.4Kg (n = 1034)**	***P value***	**HGS≥37.4Kg (n = 774)**	**HGS＜37.4Kg (n = 774)**	***P value***	**HGS≥24Kg (n = 4236)**	**HGS＜24Kg (n = 1059)**	***P value***	**HGS≥24Kg (n = 788)**	**HGS＜24Kg (n = 788)**	***P value***
handgrip, Mean ± SD	48.8 ± 7.4	31.7 ± 4.6	< 0.001	45.4 ± 6.3	32.3 ± 4.4	< 0.001	31.2 ± 4.8	20.2 ± 3.2	< 0.001	29.0 ± 3.9	20.7 ± 2.8	< 0.001
Age, Mean ± SD	43.3 ± 16.8	60.3 ± 18.8	< 0.001	56.0 ± 17.6	56.5 ± 18.9	0.606	43.5 ± 16.5	62.0 ± 18.0	< 0.001	58.2 ± 16.0	58.9 ± 18.2	0.415
BMI, Mean ± SD	28.6 ± 6.3	27.1 ± 5.8	< 0.001	27.4 ± 4.8	27.4 ± 6.1	0.95	29.6 ± 8.0	28.0 ± 6.9	< 0.001	28.7 ± 6.3	28.5 ± 7.3	0.486
Race, n (%)			< 0.001			0.682			< 0.001			0.637
Mexican American	489 (11.8)	132 (12.8)		111 (14.3)	104 (13.4)		476 (11.2)	125 (11.8)		113 (14.3)	90 (11.4)	
Other Hispanic	339 (8.2)	110 (10.6)		68 (8.8)	82 (10.6)		401 (9.5)	128 (12.1)		95 (12.1)	97 (12.3)	
Non-Hispanic White	1661 (40.1)	421 (40.7)		321 (41.5)	303 (39.1)		1656 (39.1)	458 (43.2)		300 (38.1)	321 (40.7)	
Non-Hispanic Black	1043 (25.2)	173 (16.7)		130 (16.8)	141 (18.2)		1108 (26.2)	156 (14.7)		137 (17.4)	134 (17)	
Non-Hispanic Asian	458 (11.1)	174 (16.8)		117 (15.1)	122 (15.8)		469 (11.1)	167 (15.8)		124 (15.7)	126 (16)	
Other Race including MultiRacial	151 (3.6)	24 (2.3)		27 (3.5)	22 (2.8)		126 (3)	25 (2.4)		19 (2.4)	20 (2.5)	
Education, n (%)			< 0.001			0.997			< 0.001			0.952
Less than 9th grade	253 (6.1)	168 (16.2)		99 (12.8)	97 (12.5)		206 (4.9)	155 (14.6)		89 (11.3)	86 (10.9)	
9-11th grade	644 (15.6)	160 (15.5)		119 (15.4)	122 (15.8)		584 (13.8)	172 (16.2)		118 (15)	120 (15.2)	
High school graduate or equivalent	1001 (24.2)	227 (22)		177 (22.9)	174 (22.5)		881 (20.8)	246 (23.2)		191 (24.2)	183 (23.2)	
College graduate or above	2242 (54.1)	478 (46.2)		378 (48.8)	380 (49.1)		2564 (60.5)	484 (45.7)		390 (49.5)	399 (50.6)	
other	1 (0)	1 (0.1)		1 (0.1)	1 (0.1)		1 (0)	2 (0.2)				

Marriage, n (%)			< 0.001			0.925			< 0.001			0.987
Married	2084 (50.3)	557 (53.9)		419 (54.1)	428 (55.3)		1871 (44.2)	418 (39.5)		339 (43)	353 (44.8)	
Widowed	75 (1.8)	106 (10.3)		52 (6.7)	43 (5.6)		258 (6.1)	286 (27)		144 (18.3)	145 (18.4)	
Divorced	335 (8.1)	104 (10.1)		82 (10.6)	83 (10.7)		511 (12.1)	142 (13.4)		119 (15.1)	118 (15)	
Separated	106 (2.6)	33 (3.2)		27 (3.5)	29 (3.7)		162 (3.8)	36 (3.4)		25 (3.2)	25 (3.2)	
Never married	896 (21.6)	176 (17)		137 (17.7)	140 (18.1)		867 (20.5)	103 (9.7)		94 (11.9)	86 (10.9)	
Living with partner	377 (9.1)	31 (3)		25 (3.2)	26 (3.4)		310 (7.3)	40 (3.8)		33 (4.2)	31 (3.9)	
other	268 (6.5)	27 (2.6)		32 (4.1)	25 (3.2)		257 (6.1)	34 (3.2)		34 (4.3)	30 (3.8)	
Drinking, n (%)			< 0.001			0.932			< 0.001			0.988
Never drinking	363 (9.3)	128 (13.4)		85 (11)	85 (11)		756 (19.4)	320 (32.6)		224 (28.4)	222 (28.2)	
Current drinking	3261 (83.6)	735 (77.2)		629 (81.3)	625 (80.7)		2526 (64.7)	470 (47.9)		411 (52.2)	414 (52.5)	
Ever drinking	275 (7.1)	89 (9.3)		60 (7.8)	64 (8.3)		622 (15.9)	192 (19.6)		153 (19.4)	152 (19.3)	
Smoking, n (%)			< 0.001			0.585			< 0.001			0.545
Never smoking	2052 (49.6)	467 (45.2)		359 (46.4)	340 (43.9)		2769 (65.4)	722 (68.3)		543 (68.9)	523 (66.4)	
Current smoking	1053 (25.5)	190 (18.4)		149 (19.3)	161 (20.8)		774 (18.3)	113 (10.7)		94 (11.9)	99 (12.6)	
Ever smoking	1031 (24.9)	376 (36.4)		266 (34.4)	273 (35.3)		688 (16.3)	222 (21)		151 (19.2)	166 (21.1)	
Diabetes, n (%)			< 0.001			0.846			< 0.001			0.692
No	3764 (90.9)	800 (77.4)		629 (81.3)	626 (80.9)		3842 (90.7)	834 (78.8)		646 (82)	652 (82.7)	
Yes	377 (9.1)	234 (22.6)		145 (18.7)	148 (19.1)		394 (9.3)	225 (21.2)		142 (18)	136 (17.3)	
Asthma, n (%)			0.723			0.642			0.033			0.89
No	3587 (86.6)	900 (87)		676 (87.3)	682 (88.1)		3487 (82.3)	901 (85.1)		664 (84.3)	666 (84.5)	
Yes	554 (13.4)	134 (13)		98 (12.7)	92 (11.9)		749 (17.7)	158 (14.9)		124 (15.7)	122 (15.5)	
Congestive heart failure, n (%)			< 0.001			0.91			< 0.001			0.7
No	4075 (98.4)	943 (91.2)		732 (94.6)	733 (94.7)		4163 (98.3)	981 (92.6)		755 (95.8)	758 (96.2)	

Yes	66 (1.6)	91 (8.8)		42 (5.4)	41 (5.3)		73 (1.7)	78 (7.4)		33 (4.2)	30 (3.8)	
Coronary heart disease, n (%)			< 0.001			0.46			< 0.001			0.321
No	4023 (97.2)	920 (89)		706 (91.2)	714 (92.2)		4171 (98.5)	990 (93.5)		750 (95.2)	758 (96.2)	
Yes	118 (2.8)	114 (11)		68 (8.8)	60 (7.8)		65 (1.5)	69 (6.5)		38 (4.8)	30 (3.8)	
Stroke, n (%)			< 0.001			0.735			< 0.001			0.347
No	4060 (98)	956 (92.5)		731 (94.4)	734 (94.8)		4152 (98)	963 (90.9)		746 (94.7)	754 (95.7)	
Yes	81 (2)	78 (7.5)		43 (5.6)	40 (5.2)		84 (2)	96 (9.1)		42 (5.3)	34 (4.3)	
Cancer, n (%)			< 0.001			0.817			< 0.001			0.619
No	3882 (93.7)	883 (85.4)		676 (87.3)	679 (87.7)		3950 (93.2)	869 (82.1)		668 (84.8)	675 (85.7)	
Yes	259 (6.3)	151 (14.6)		98 (12.7)	95 (12.3)		286 (6.8)	190 (17.9)		120 (15.2)	113 (14.3)	
High blood pressure, n (%)			< 0.001			0.837			< 0.001			0.88
No	2875 (69.4)	564 (54.5)		446 (57.6)	450 (58.1)		2961 (69.9)	466 (44)		390 (49.5)	393 (49.9)	
Yes	1266 (30.6)	470 (45.5)		328 (42.4)	324 (41.9)		1275 (30.1)	593 (56)		398 (50.5)	395 (50.1)	
All-cause mortality, n (%)			< 0.001			< 0.001			< 0.001			< 0.001
No	3953 (95.5)	753 (72.8)		704 (91)	597 (77.1)		4095 (96.7)	822 (77.6)		740 (93.9)	648 (82.2)	
Yes	188 (4.5)	281 (27.2)		70 (9)	177 (22.9)		141 (3.3)	237 (22.4)		48 (6.1)	140 (17.8)	
Heart mortality, n (%)			< 0.001			0.002			< 0.001			< 0.001
No	4100 (99)	959 (92.7)		756 (97.7)	732 (94.6)		4204 (99.2)	985 (93)		780 (99)	749 (95.1)	
Yes	41 (1)	75 (7.3)		18 (2.3)	42 (5.4)		32 (0.8)	74 (7)		8 (1)	39 (4.9)	
Follow-up time, Mean ± SD	83.0 ± 16.0	73.8 ± 24.4	< 0.001	82.1 ± 17.2	77.0 ± 22.9	< 0.001	82.8 ± 15.3	75.6 ± 22.8	< 0.001	81.6 ± 15.7	78.7 ± 21.3	0.002
